# Supplementary material for: The human primary visual cortex (V1) encodes the perceived position of static but not moving objects
Source: Commun Biol. 2022 Mar 1;5:181. doi: 10.1038/s42003-022-03136-y (PMC8888673; doi:10.1038/s42003-022-03136-y)
Supplement: Supplementary file 2 — Supplementary Materials [file 42003_2022_3136_MOESM2_ESM.pdf]

Supplementary Table 1. Two-way repeated-measures ANOVA for Gaussian fit parameters

		Condition			Location			Condition × Location		
	df	F	p	df	F	p	df	F	p	Sig. level†
	(1, 7)			(1, 7)			(1, 7)			
Peak location (μ)		2.58	0.152		0.63	0.454		3.35	0.110	0.05
Baseline (α)		3.40	0.108		1.43	0.271		0.65	0.447	0.0125
Amplitude (β)		3.43	0.107		1.45	0.268		0.66	0.444	0.0125
Spread (σ)		3.61	0.099		1.06	0.338		0.01	0.928	0.0125
Goodness-of-fit (R^2)		0.02	0.907		0.09	0.772		0.54	0.488	0.0125

† Note that for parameters without *a priori* hypotheses (baseline, response amplitude, spread and goodness-of-fit), the significance level has been Bonferroni-corrected for multiple comparisons (corrected level at 0.05/4 = 0.0125).

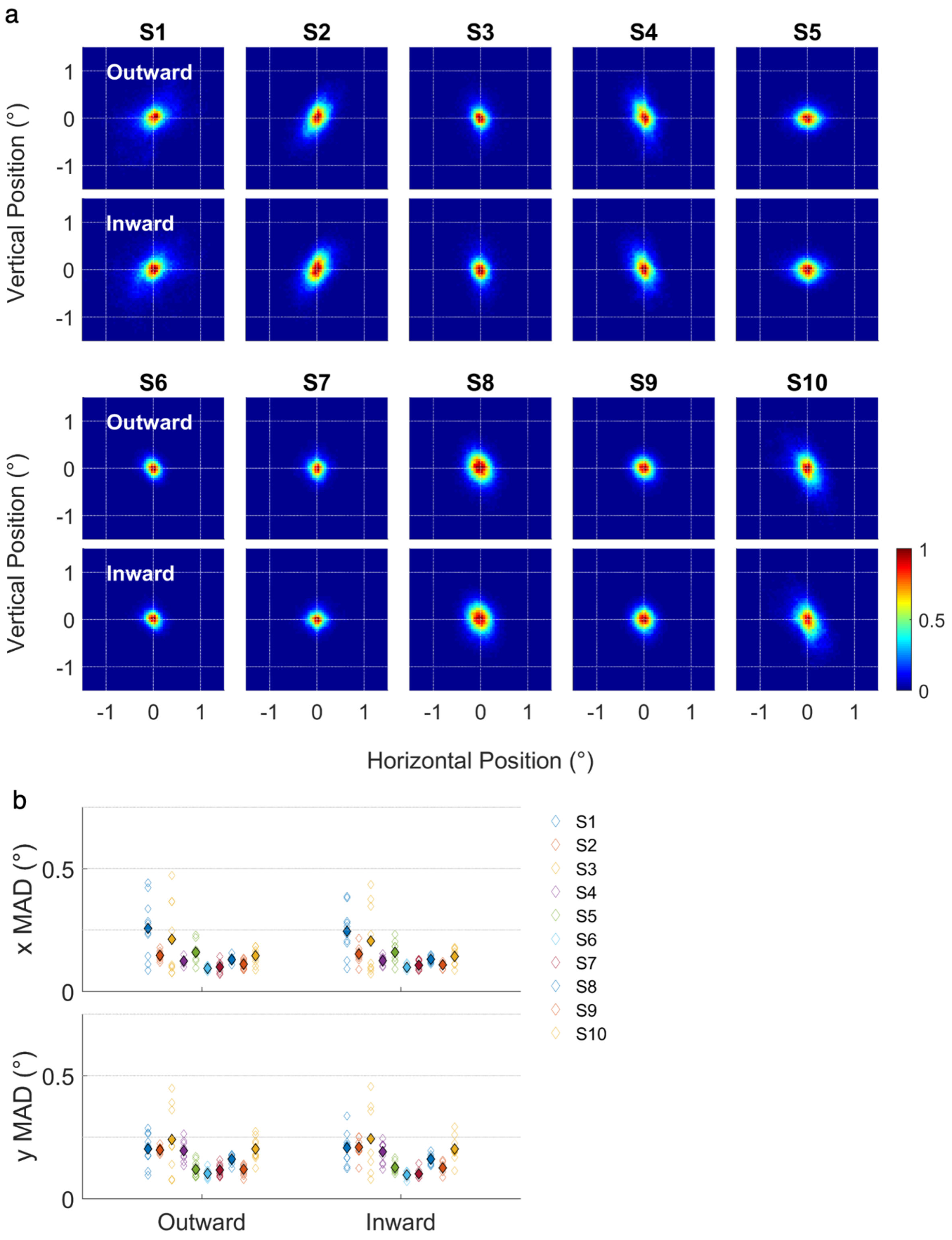

Supplementary Figure 1. Muller-Lyer eyetracking analyses for each participant. (a) Eye position distribution visualised as 2D histograms for each condition. (b) Median absolute deviation (MAD) for the horizontal and vertical eye positions across runs; filled diamonds denote averaged MAD across runs.

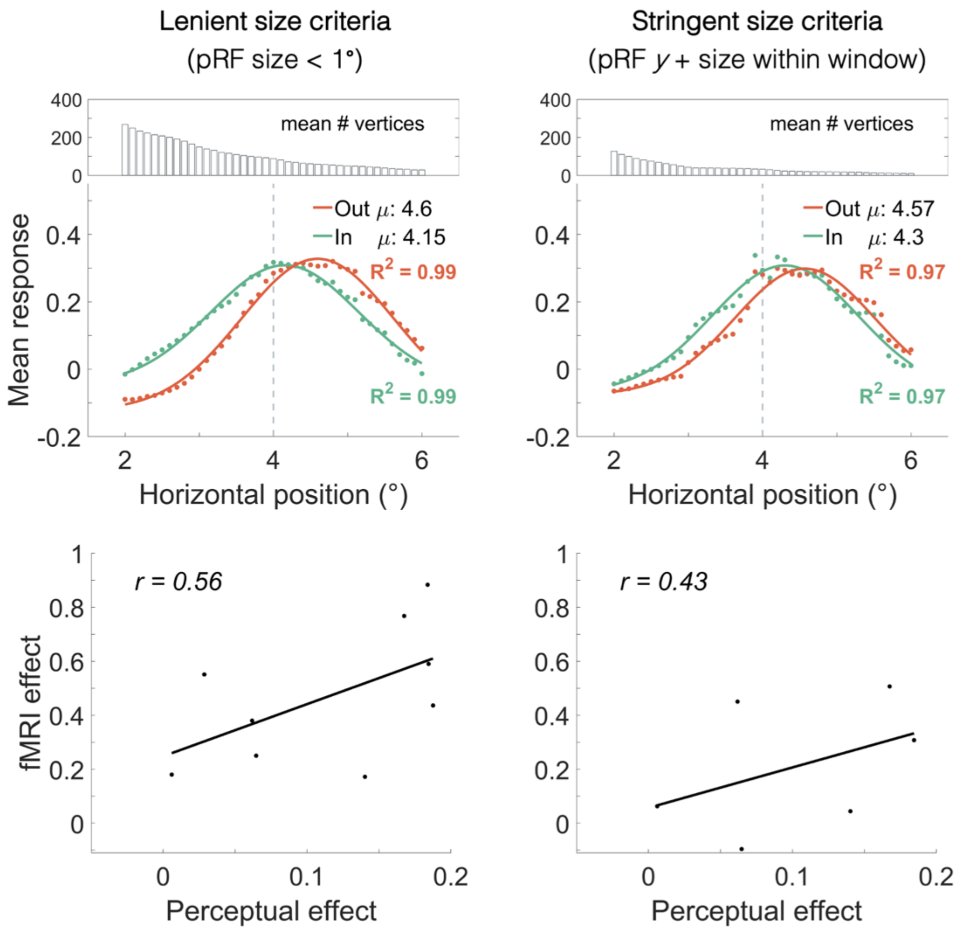

Supplementary Figure 2. Muller-Lyer experiment control analyses. Shift in neural signature was reduced after applying a more stringent sampling criterion, excluding pRFs with significant overlap with the contextual stimuli.

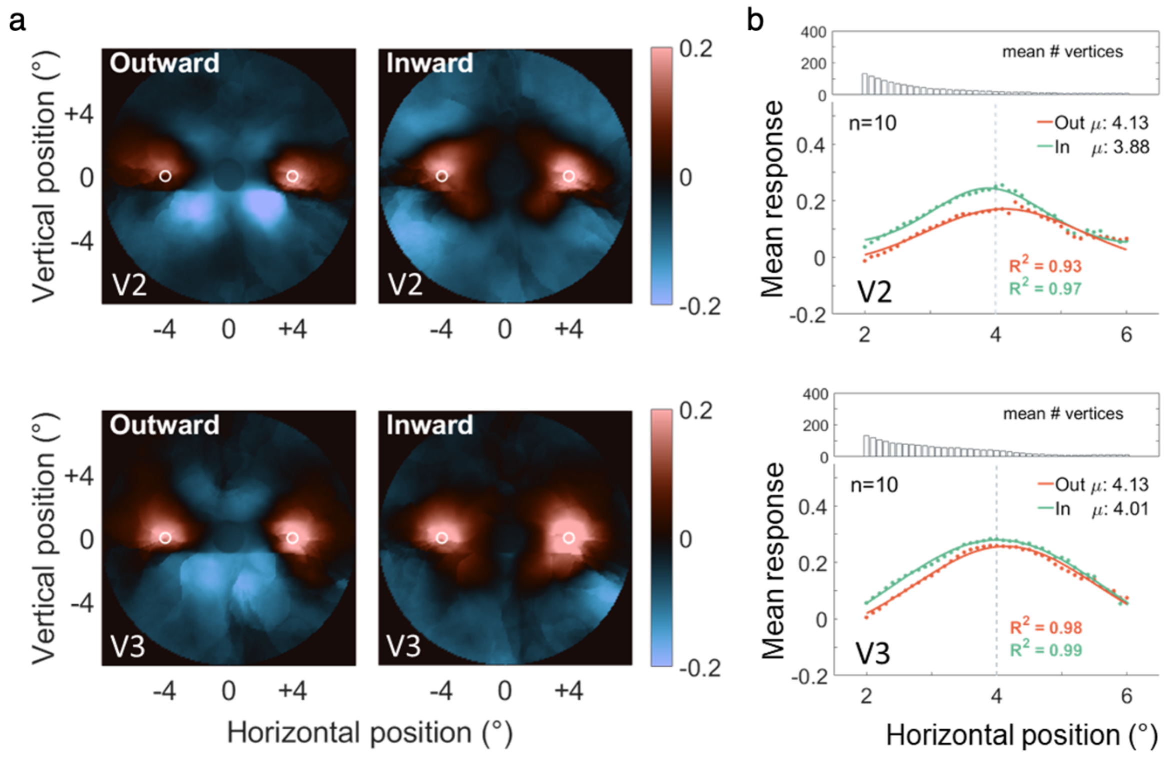

Supplementary Figure 3. Muller-Lyer data from V2 and V3. (a) Brain responses reconstructed in visual field. White circles denote the physical target locations. (b) Group-level neural signature collapsed across hemifields and fit with a Gaussian function. The vertical dotted line denotes physical target location. Consistent with the illusion, targets with outward fins appeared more peripheral. Top row: V2. Bottom row: V3.

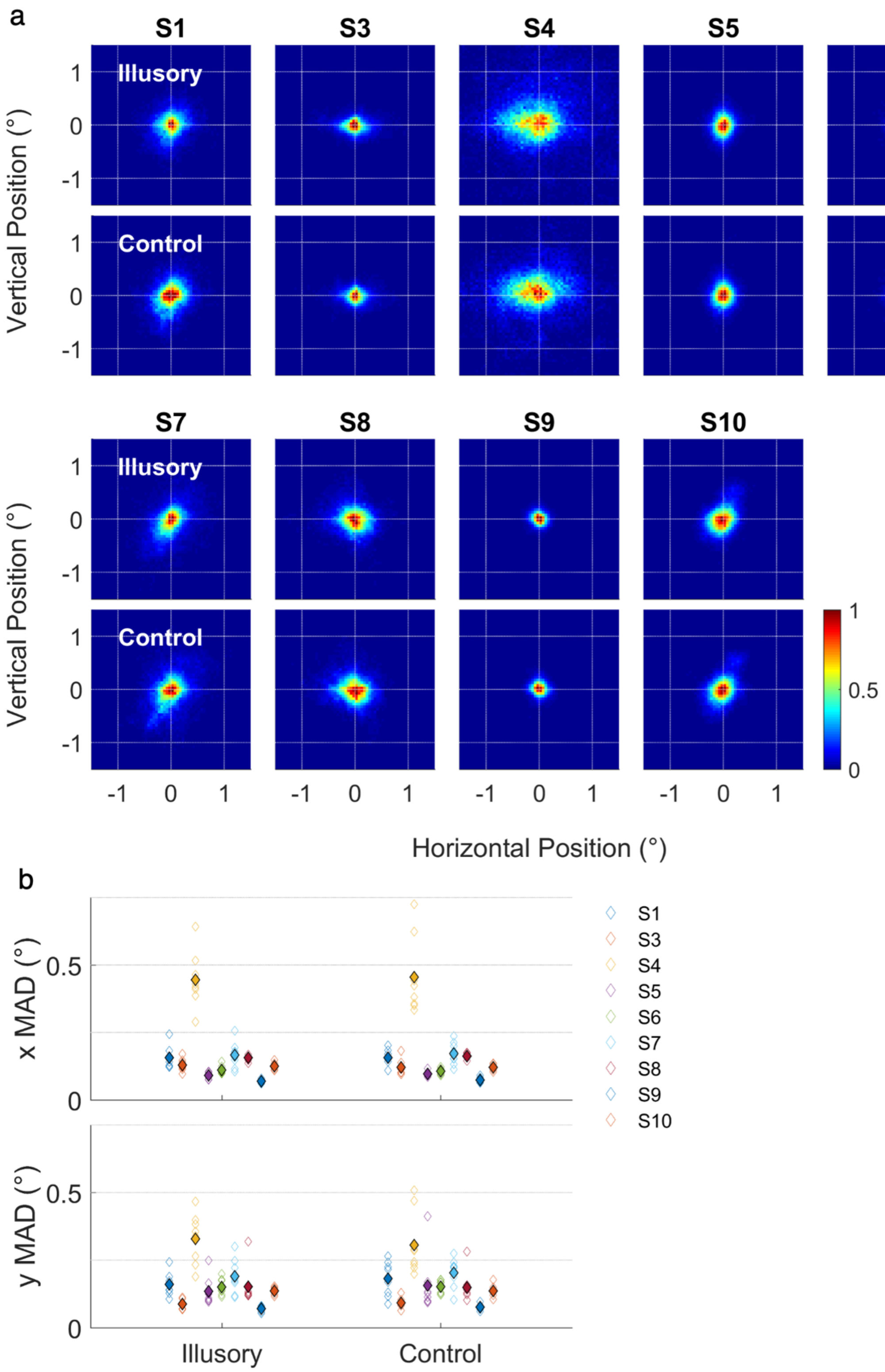

Supplementary Figure 4. Curveball experiment eyetracking analyses for nine out of ten participants (no eyetracking data were collected on S2 due to technical issues on the day of the scan). (a) Eye position distribution visualised as 2D histograms for each condition. (b) MAD for the horizontal and vertical eye positions across runs; filled diamonds denote averaged MAD across runs.

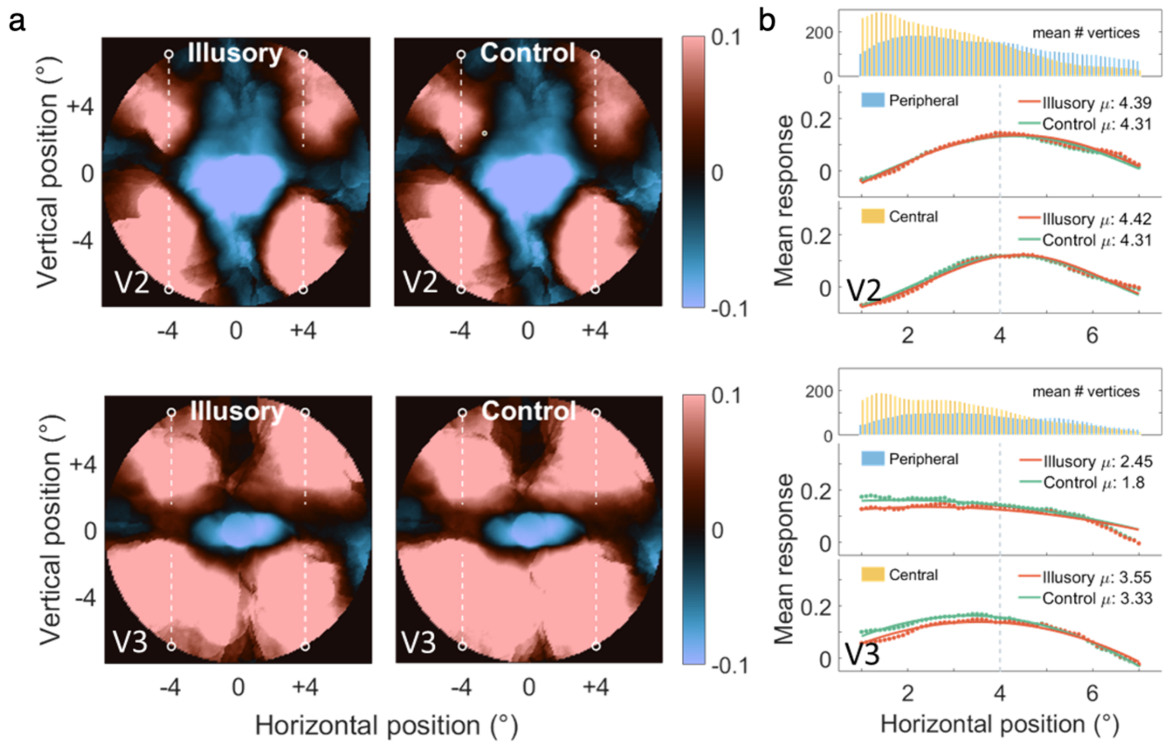

Supplementary Figure 5. Curveball data from V2 and V3. (a) Brain responses reconstructed in visual field. White dashed lines denote the physical motion paths. (b) Group-level neural signatures collapsed across hemifields and fit with a Gaussian function. Both illusory and control signatures across both sliding window locations were centered on the physical motion path (vertical dotted lines). Top row: V2. Bottom row: V3.
