# Supplementary material for: The human primary visual cortex (V1) encodes the perceived position of static but not moving objects
Source: Commun Biol. 2022 Mar 1;5:181. doi: 10.1038/s42003-022-03136-y (PMC8888673; doi:10.1038/s42003-022-03136-y)
Supplement: Supplementary file 3 — Description of Additional Supplementary Files [file 42003_2022_3136_MOESM3_ESM.pdf]

Description of Additional Supplementary Files

File name: Supplementary Movie 1
Description: Dot variant of the Muller-Lyer illusion: outward-facing.

File name: Supplementary Movie 2
Description: Dot variant of the Muller-Lyer illusion: inward-facing.

File name: Supplementary Movie 3
Description: Variant of the curveball illusion comprising four Gabor patches (control).

File name: Supplementary Movie 4
Description: Variant of the curveball illusion comprising four Gabor patches (illusory).
