# Supplementary figures and images for: The human primary visual cortex (V1) encodes the perceived position of static but not moving objects
Source: Commun Biol. 2022 Mar 1;5:181. doi: 10.1038/s42003-022-03136-y (PMC8888673; doi:10.1038/s42003-022-03136-y)

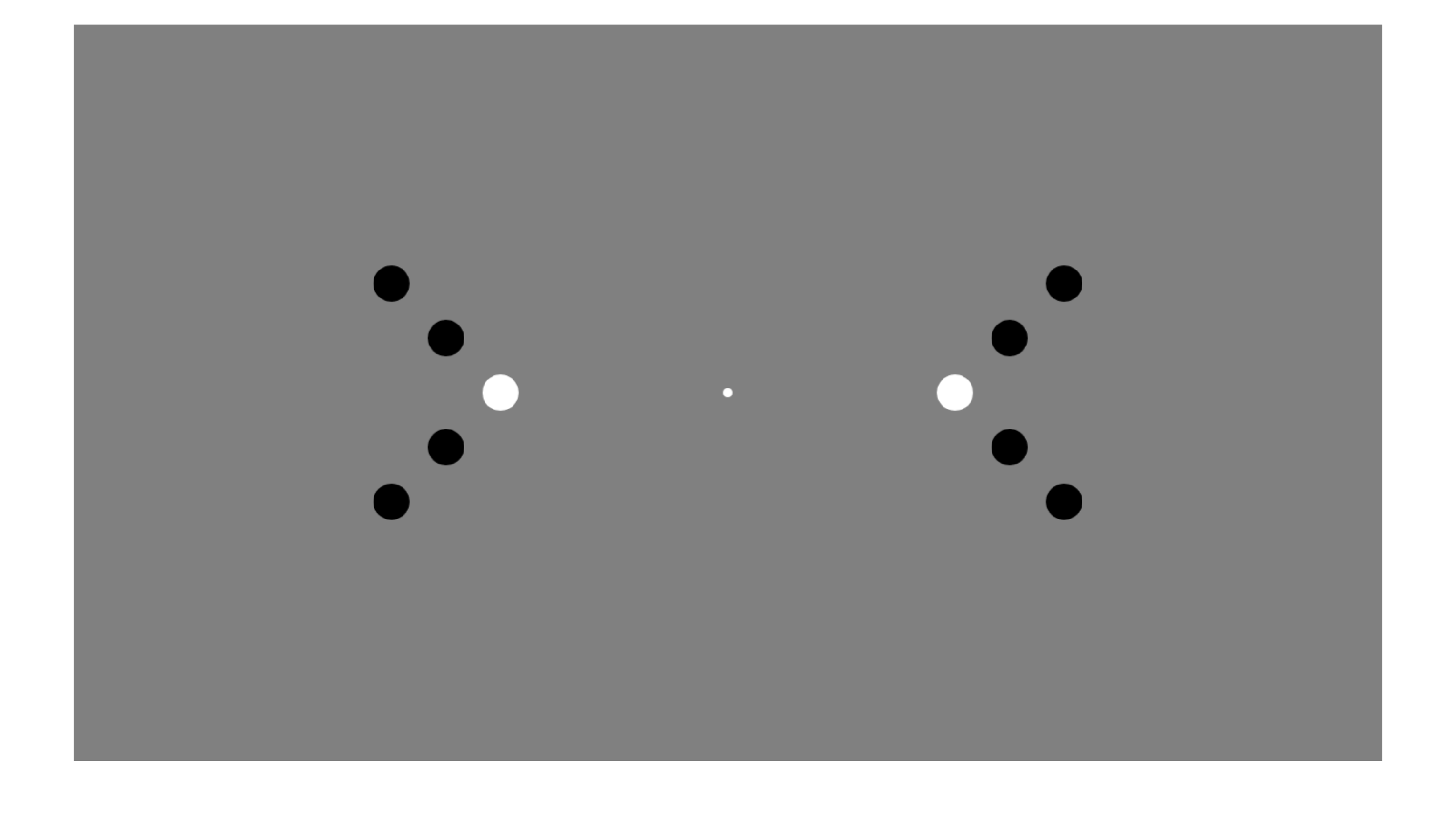

Supplement: Supplementary file 4 — Supplementary Movie 1 [file 42003_2022_3136_MOESM4_ESM.gif]

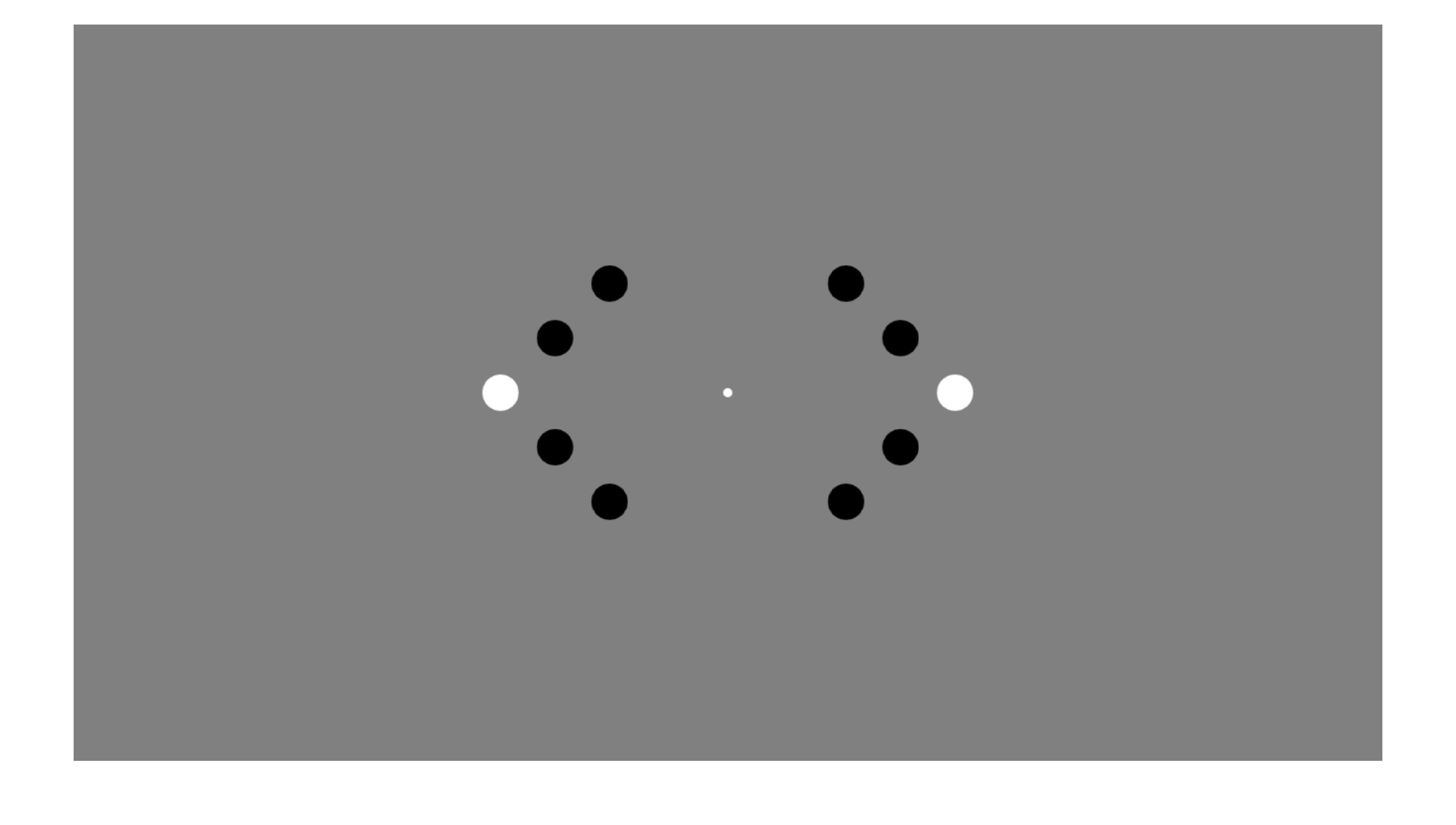

Supplement: Supplementary file 5 — Supplementary Movie 2 [file 42003_2022_3136_MOESM5_ESM.gif]

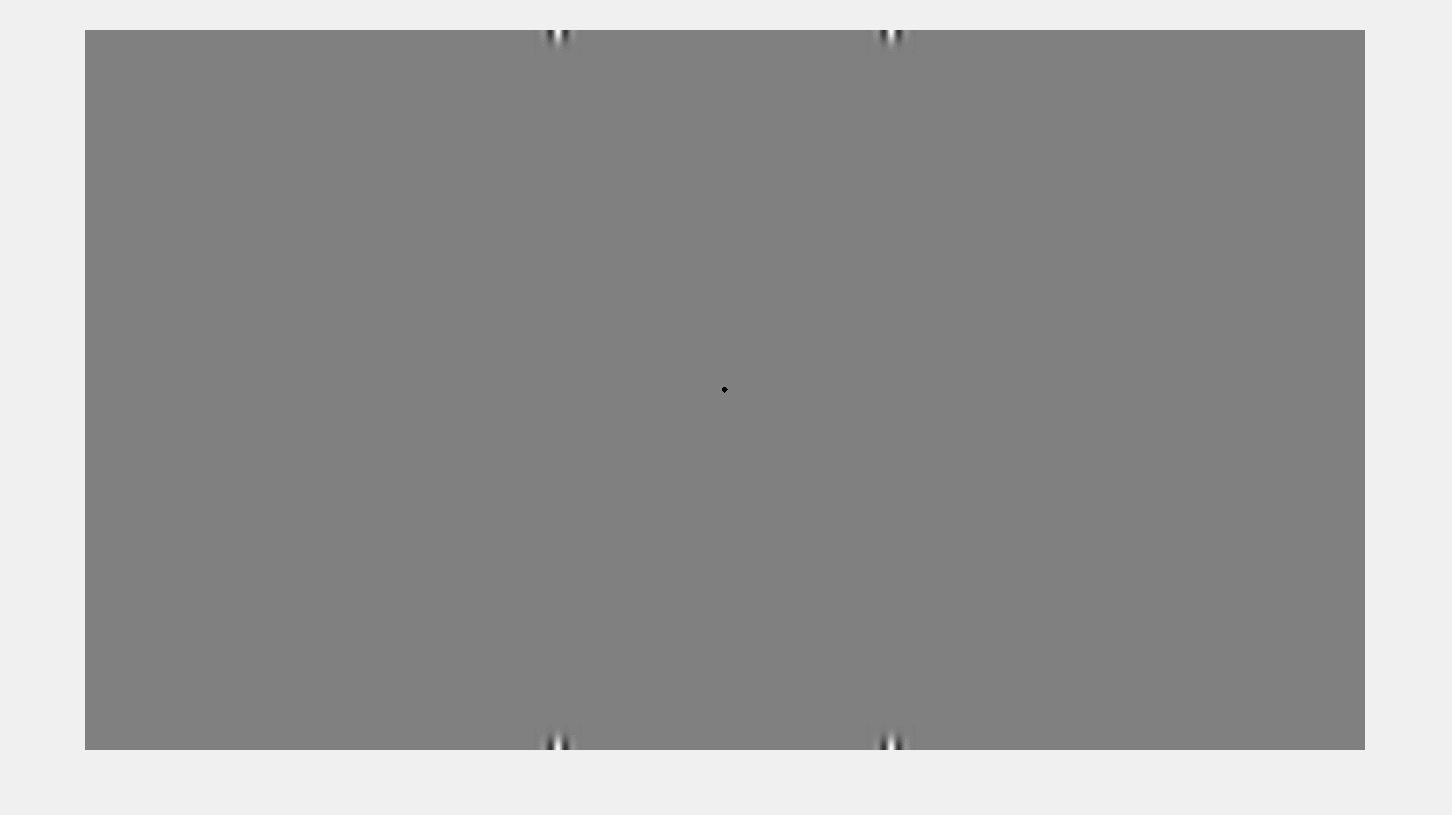

Supplement: Supplementary file 6 — Supplementary Movie 3 [file 42003_2022_3136_MOESM6_ESM.gif]

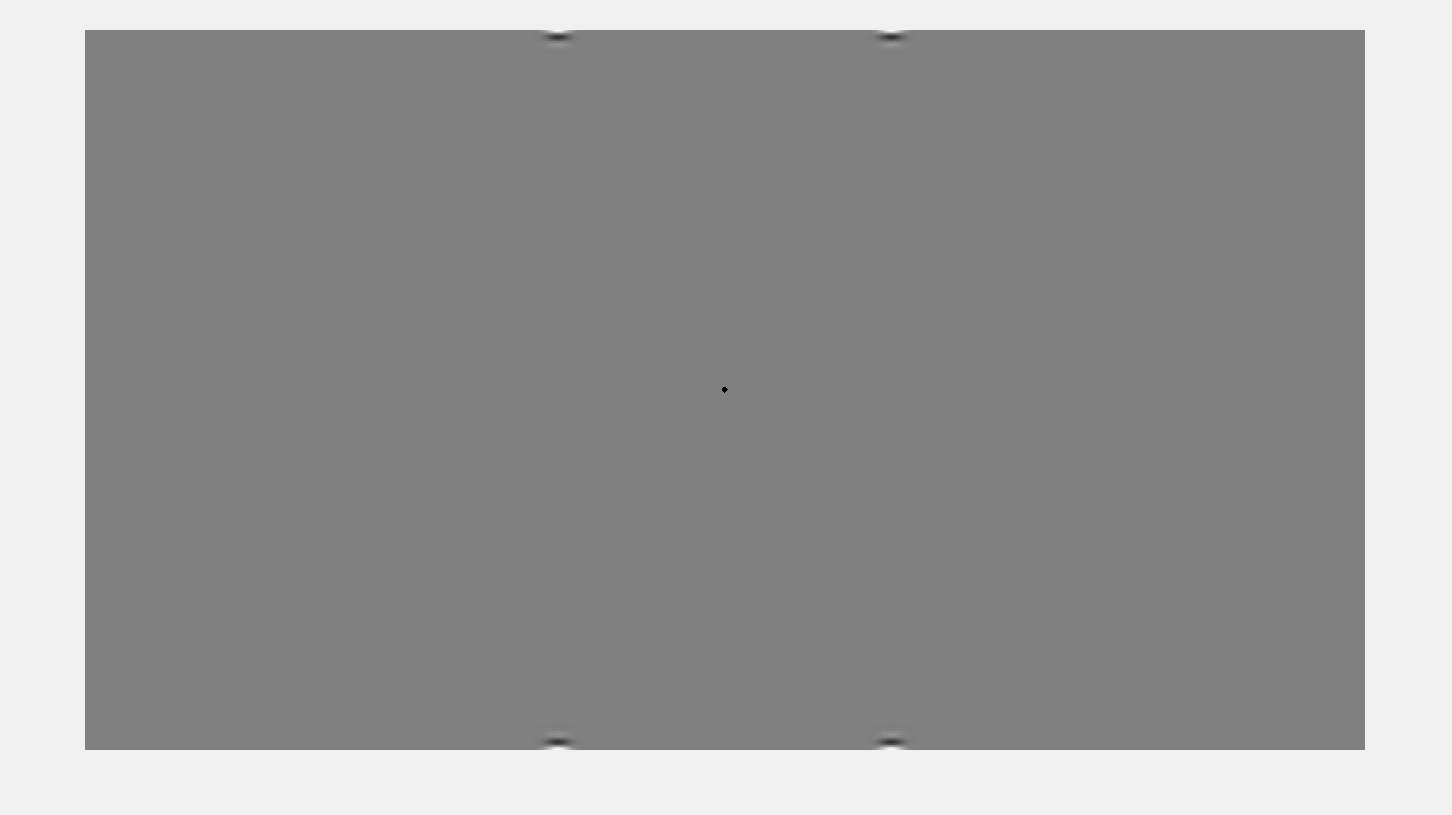

Supplement: Supplementary file 7 — Supplementary Movie 4 [file 42003_2022_3136_MOESM7_ESM.gif]
