# Supplementary material for: The human primary visual cortex (V1) encodes the perceived position of static but not moving objects
Source: Commun Biol. 2022 Mar 1;5:181. doi: 10.1038/s42003-022-03136-y (PMC8888673; doi:10.1038/s42003-022-03136-y)
Supplement: Supplementary file 8 — Reporting summary [file 42003_2022_3136_MOESM8_ESM.pdf]

nature portfolio

Corresponding author(s): Man-Ling Ho
D. Samuel Schwarzkopf

Last updated by author(s): Dec 31, 2021

Reporting Summary

Nature Portfolio wishes to improve the reproducibility of the work that we publish. This form provides structure for consistency and transparency in reporting. For further information on Nature Portfolio policies, see our Editorial Policies and the Editorial Policy Checklist.

Statistics

For all statistical analyses, confirm that the following items are present in the figure legend, table legend, main text, or Methods section.

n/a	Confirmed	
☐	☒	The exact sample size (*n*) for each experimental group/condition, given as a discrete number and unit of measurement
☐	☒	A statement on whether measurements were taken from distinct samples or whether the same sample was measured repeatedly
☐	☒	The statistical test(s) used AND whether they are one- or two-sided<br>*Only common tests should be described solely by name; describe more complex techniques in the Methods section.*
☐	☒	A description of all covariates tested
☐	☒	A description of any assumptions or corrections, such as tests of normality and adjustment for multiple comparisons
☐	☒	A full description of the statistical parameters including central tendency (e.g. means) or other basic estimates (e.g. regression coefficient) AND variation (e.g. standard deviation) or associated estimates of uncertainty (e.g. confidence intervals)
☐	☒	For null hypothesis testing, the test statistic (e.g. *F*, *t*, *r*) with confidence intervals, effect sizes, degrees of freedom and *P* value noted<br>*Give P values as exact values whenever suitable.*
☒	☐	For Bayesian analysis, information on the choice of priors and Markov chain Monte Carlo settings
☒	☐	For hierarchical and complex designs, identification of the appropriate level for tests and full reporting of outcomes
☐	☒	Estimates of effect sizes (e.g. Cohen's *d*, Pearson's *r*), indicating how they were calculated

Our web collection on statistics for biologists contains articles on many of the points above.

Software and code

Policy information about availability of computer code

Data collection	MATLAB R2014a (Version 8.3; The MathWorks Inc., 2014); Psychtoolbox (Version 3.0.11; Brainard, 1997)
Data analysis	SPM12 (Version 6685; Wellcome Trust Centre for NeuroImaging); FreeSurfer (Version 5.3; Dale, Fischl and Sereno, 1999; Fischl, Sereno and Dale, 1999); SamSrf Toolbox Version 5.84 for pRF analysis and Version 6.19 for back-projection; https://osf.io/mrzqy/)

For manuscripts utilizing custom algorithms or software that are central to the research but not yet described in published literature, software must be made available to editors and reviewers. We strongly encourage code deposition in a community repository (e.g. GitHub). See the Nature Portfolio guidelines for submitting code & software for further information.

Data

Policy information about availability of data

All manuscripts must include a data availability statement. This statement should provide the following information, where applicable:

- Accession codes, unique identifiers, or web links for publicly available datasets
- A description of any restrictions on data availability
- For clinical datasets or third party data, please ensure that the statement adheres to our policy

All data and code necessary for reproducing the figures are publicly available on OSF (https://osf.io/5qxab). Full data sets cannot be shared publicly due to data protection requirements but may be shared by request.

Field-specific reporting

Please select the one below that is the best fit for your research. If you are not sure, read the appropriate sections before making your selection.

☐ Life sciences ☒ Behavioural & social sciences ☐ Ecological, evolutionary & environmental sciences

For a reference copy of the document with all sections, see nature.com/documents/nr-reporting-summary-flat.pdf

Behavioural & social sciences study design

All studies must disclose on these points even when the disclosure is negative.

Study description	Quantitative experimental
Research sample	Ten participants (five females; age range 23–49 years; two left-handed) took part in the Muller-Lyer experiment, including one of the authors. Ten participants (two from the Muller-Lyer experiment; seven females; age range 20–48 years; two left-handed) took part in the Curveball experiment. Participants were either UCL researchers or were recruited online via the UCL psychology subject pool.
Sampling strategy	Convenience sampling procedure. Sample size was determined based on similar studies on size representation (e.g. Murray et al., 2006; Fang et al., 2008; He et al., 2015), which found evidence that V1 activity correlated with perceptual effect using a relatively small sample size (n<10).
Data collection	Participants were placed inside a MRI scanner with the visual stimuli backprojected onto a screen at the back of the bore, and viewed through a mirror mounted on the head coil. Eye movements were monitored using an MR-compatible SR Research EyeLink 1000 eye tracker (Ottawa, CA). The researcher was present, although this was a within-subject design and the experimental condition was controlled by the computer.
Timing	Data for the Muller-Lyer experiment was collected between August-October 2017; Curveball experiment was between March-April 2018. For a few participants, we generated visual field maps based on data collected in 2015/2016.
Data exclusions	No data was excluded
Non-participation	No participants dropped out
Randomization	Not allocated into experimental groups; within-subject design

Reporting for specific materials, systems and methods

We require information from authors about some types of materials, experimental systems and methods used in many studies. Here, indicate whether each material, system or method listed is relevant to your study. If you are not sure if a list item applies to your research, read the appropriate section before selecting a response.

Materials & experimental systems

n/a	Involved in the study	
☒	☐	Antibodies
☒	☐	Eukaryotic cell lines
☒	☐	Palaeontology and archaeology
☒	☐	Animals and other organisms
☐	☒	Human research participants
☒	☐	Clinical data
☒	☐	Dual use research of concern

Methods

n/a	Involved in the study	
☒	☐	ChIP-seq
☒	☐	Flow cytometry
☐	☒	MRI-based neuroimaging

Human research participants

Policy information about studies involving human research participants

Population characteristics	See above
Recruitment	Participants were either UCL researchers or were recruited online via the UCL psychology subject pool, and were known to be cooperative. These are unlikely to be representative of the general population.
Ethics oversight	University College London Research Ethics Committee

Note that full information on the approval of the study protocol must also be provided in the manuscript.

Magnetic resonance imaging

Experimental design

Design type	Task; block-design (main experiment), event-related (pRF mapping)
Design specifications	Muller-Lyer: Stimuli were presented in blocks each comprising 10s presentation of background-only period, 16s of illusory period, followed by a 6s fixation-only period. Each run contained a total of eight blocks, where each condition appeared four times. Participants completed ten runs of the experiment; 266 volumes per run.<br><br>Curveball: Each run started with an initial 15s fixation-only period, followed by six repeats of a 15s stimulus period, a 1s fixation-only interval, another 15s stimulus period and a final 15s fixation-only period. Participants completed eight runs of the experiment; 301 volumes per run.<br><br>Retinotopic (pRF) mapping: The mapping procedure involved the simultaneous presentation of a rotating wedge and an expanding–contracting ring. The wedge aperture (12 degree polar angle) rotated around a black central fixation dot either clockwise or counter-clockwise in 60 discrete steps (1 step/s), while a ring aperture expanded or contracted in 36 logarithmic steps (1 step/s). Each mapping run had a total of three cycles of wedge rotation and five cycles of ring expansion-contraction and contained a total of four blocks (block 1: clockwise, expansion; block 2: clockwise, contraction; block 3: counter-clockwise, expansion; block 4: counter-clockwise, contraction). Each block began with 90s of stimulus presentation (1.5 cycles of wedge rotation; 2.5 cycles of ring expansion or contraction) followed by a 30s fixation-only interval. Within each block, image type alternated between intact and phase-scrambled every 15s. Participants completed three runs; 490 volumes per run.
Behavioral performance measures	Eyetracking and button presses during the retinotopic mapping and main fMRI experiment

Acquisition

Imaging type(s)	Functional; structural
Field strength	1.5
Sequence & imaging parameters	All functional and anatomical images were acquired on a Siemens Avanto 1.5T MRI scanner with a customised 30-channel head coil (32-channel with two anterior channels removed to avoid restriction of view). Functional images were collected using T2*-weighted multi-band 2D echo-planar imaging sequence 2 centred around the occipital cortex (TR = 1000ms, TE = 55ms, flip angle = 75 degree, 36 transverse slices, acceleration factor = 4, FOV = 96 × 96 voxels) at a resolution of 2.3mm isotropic voxels.
Area of acquisition	Slices were tilted to be approximately parallel to the calcarine sulcus to ensure coverage of the occipital cortex, and the occipital-temporal and inferior parietal cortices

Diffusion MRI ☐ Used ☒ Not used

Preprocessing

Preprocessing software	SPM 12 with default parameters. All functional images were preprocessed with SPM12 using default parameters (Version 6685; Wellcome Trust Centre for NeuroImaging). The images were bias-corrected for intensity inhomogeneities, realigned, unwarped and co-registered to the high-resolution anatomical scan.
Normalization	The fMRI time series for each voxel were linearly detrended and z-score normalised; these were averaged across runs for the retinotopic mapping procedure and concatenated across runs for the main experiment.
Normalization template	No spatial normalisation was done. All analysis was in native brain space.
Noise and artifact removal	Motion regressors were incorporated into the general linear model
Volume censoring	No volume censoring

Statistical modeling & inference

Model type and settings	Mass univariate. Subject-wise analysis using general linear model, where boxcar regressors were defined for each of the conditions and convolved with a canonical HRF (de Haas et al., 2014). Six motion regressors and a global covariate were also included in the general linear model. Signal for the target was isolated by contrasting baseline (Muller-Lyer: background-only periods; Curveball: fixation-only periods) with the stimulus-of-interest periods for each of the condition.
Effect(s) tested	Whether V1 activity differed spatially between the stimulus conditions (Muller-Lyer: outward versus inward arrows; Curveball: illusory versus control)

Specify type of analysis: ☐ Whole brain ☒ ROI-based ☐ Both

Anatomical location(s)	V1 was delineated based on smoothed polar angle, eccentricity and field sign maps using the SamSrf toolbox. The reversals of polar angle indicated the boundaries between V1 and V2 (Sereno et al., 1995). V1 was delineated as full hemifield maps within the calcarine sulcus.
Statistic type for inference (See Eklund et al. 2016)	Voxel-wise
Correction	N/A

Models & analysis

n/a	Involved in the study
☒	☐ Functional and/or effective connectivity
☒	☐ Graph analysis
☒	☐ Multivariate modeling or predictive analysis
